# Acrolein acts as a neurotoxin in the nigrostriatal dopaminergic system of rat: involvement of α-synuclein aggregation and programmed cell death

**DOI:** 10.1038/srep45741

**Published:** 2017-04-12

**Authors:** Yi-Ting Wang, Hui-Ching Lin, Wei-Zhong Zhao, Hui-Ju Huang, Yu-Li Lo, Hsiang-Tsui Wang, Anya Maan-Yuh Lin

**Affiliations:** 1 Institute of Physiology, National Yang Ming University, Taipei, Taiwan; 2 Institute of Pharmacology, National Yang Ming University, Taipei, Taiwan; 3 Department of Medical Research, Taipei Veterans General Hospital, Taipei, Taiwan; 4 Faculty of Pharmacy, National Yang-Ming University, Taipei, Taiwan

## Abstract

Clinical studies report significant increases in acrolein (an α,β-unsaturated aldehyde) in the substantia nigra (SN) of patients with Parkinson’s disease (PD). In the present study, acrolein-induced neurotoxicity in the nigrostriatal dopaminergic system was investigated by local infusion of acrolein (15, 50, 150 nmoles/0.5 μl) in the SN of Sprague-Dawley rats. Acrolein-induced neurodegeneration of nigrostriatal dopaminergic system was delineated by reductions in tyrosine hydroxylase (TH) levels, dopamine transporter levels and TH-positive neurons in the infused SN as well as in striatal dopamine content. At the same time, apomorphine-induced turning behavior was evident in rats subjected to a unilateral infusion of acrolein in SN. Acrolein was pro-oxidative by increasing 4-hydroxy-2-nonenal and heme oxygenase-1 levels. Furthermore, acrolein conjugated with proteins at lysine residue and induced α-synuclein aggregation in the infused SN. Acrolein was pro-inflammatory by activating astrocytes and microglia. In addition, acrolein activated caspase 1 in the infused SN, suggesting acrolein-induced inflammasome formation. The neurotoxic mechanisms underlying acrolein-induced neurotoxicity involved programmed cell death, including apoptosis and necroptosis. Compared with well-known Parkinsonian neurotoxins, including 1-methyl-4-phenyl-1,2,3,6-tetrahydropyridine and rotenone which do not exist in the SN of PD patients, our *in vivo* study shows that acrolein acts as a Parkinsonian neurotoxin in the nigrostriatal dopaminergic system of rat brain.

Acrolein, an α,β-unsaturated aldehyde, is known as a toxin exogenously produced from environmental pollution, including tobacco smoking^1^, incomplete combustion of plastic materials^2^ and cooking fumes^3^. Endogenously, acrolein is produced from lipid peroxidation of polyunsaturated fatty acid, DNA and proteins^4^,^5^,^6^ as well as metabolism of allyl compounds^7^. Clinical studies have reported significant acrolein levels in brain and spinal cord of patients with central nervous system (CNS) neurodegenerative diseases, including Parkinson’s disease (PD)^8^, Alzheimer’s disease^9^ and spinal cord injury^10^. *In vitro* studies have demonstrated the neurotoxic effects of acrolein on HT22 hippocampal cells^11^, primary cortical neurons^12^,^13^ and dorsal root ganglionic neurons^13^, suggesting that acrolein plays a neurotoxic role in the CNS neurodegeneration^14^,^15^.

Oxidative stress is known to be involved in the acrolein-induced cytotoxicity. For example, acrolein is capable of initiating lipid peroxidation^6^. Furthermore, acrolein attacks mitochondrial membranes and produces reactive oxygen species (ROS)^16^. Due to its electrophilic activity, acrolein reportedly reacts with DNA^17^ and proteins^4^,^5^. A pathological role of acrolein-protein conjugation has been suggested by altering protein conformation and promoting protein aggregation in a cell-free model^18^. To support this notion, several *in vitro* studies have employed rotenone (a Parkinsonian neurotoxin) to demonstrate acrolein modified proteins, α-synuclein misfolding and aggregation in SH-SY5Y and PC12 cells^19^,^20^,^21^. Clinically, accumulation of acrolein-α-synuclein adducts was detected in the nigral dopaminergic neurons of PD patients^8^. So far, no *in vivo* studies have supported the influence of acrolein on α-synuclein aggregation in the brain of PD patients^22^.

A vicious cycle of oxidative stress, protein aggregation and cell death has been proposed for CNS neurodegeneration; this vicious cycle may be responsible for the acrolein-induced neurotoxicity in the nigrostriatal dopaminergic system, the most affected nervous system of PD. Along with the above-mentioned evidence, *in vitro* studies have demonstrated acrolein-induced necrosis, which may be due to attacking mitochondria, reducing ATP formation^16^ and increasing calpain activity^13^,^16^. Furthermore, acrolein-induced apoptosis was verified by activating caspase 3 and caspase 7 as well as forming DNA laddering, biomarkers of apoptosis^23^,^24^. Many *in vivo* studies have demonstrated the acrolein-induced neurotoxicity in Alzheimer’s disease and spinal cord injury^10^,^25^,^26^. In contrast, few *in vivo* studies have focused on the neurotoxic mechanisms of acrolein in the etiology of Parkinsonism. In the present study, intranigral infusion of acrolein was employed to mimic the elevated acrolein levels in the SN of PD patients^8^. The neurotoxic mechanisms of acrolein in nigrostriatal dopaminergic system was investigated by measuring involvement of oxidative stress, interaction of acrolein with proteins and α-synuclein, neuroinflammation and programmed cell death.

## Results

### Acrolein-induced neurodegeneration of nigrostriatal dopaminergic system

**Figure 1:**
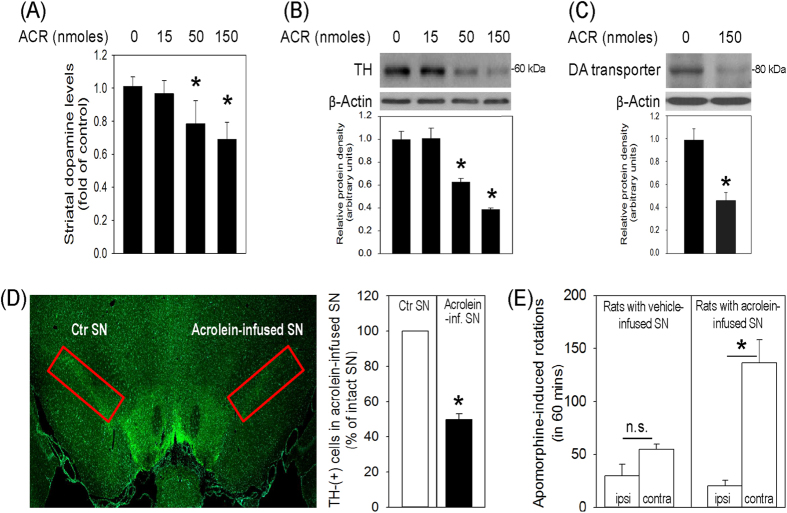
Intranigral infusion of acrolein induced neurodegeneration of the nigrostriatal dopaminergic system of rat brain. Acrolein (ARC, 15–150 nmoles) was locally infused in the substantia nigra (SN) and rats were sacrificed 7 days after intranigral infusion of acrolein. (**A**) Striatal dopamine content was measured using HPLC-ECD. Values are the mean ± S.E.M. (n = 5–6/group) *p < 0.05 in the striatum ipsilateral to acrolein-infused SN compared with that to the intact SN by one-way analysis of variance (one-way ANOVA) and followed by the LSD test as a post-hoc analysis. (**B**) Tyrosine hydroxylase (TH) in the SN was measured using Western blot. Each lane contained 3 μg proteins for all experiments. Graphs show statistic results from relative optical density of bands on the blots. Values are the mean ± S.E.M. (n = 3/group). *p < 0.05 in the acrolein-infused SN compared with the intact SN by one-way analysis of variance (one-way ANOVA) and followed by the LSD test as a post-hoc analysis. (**C**) Dopamine transporter in the SN was measured using Western blot. Each lane contained 25 μg proteins for all experiments. Values are the mean ± S.E.M. (n = 7/group). *p < 0.05 in the acrolein-infused SN compared with the intact SN by *t*-test. (**D**) Representative confocal microscopic data showed TH-positive neurons in the SN of rat subjected to intranigral infusion of acrolein (150 nmoles). TH-positive cells in acrolein-infused SN were counted and expressed as % of that in the contralateral intact SN. Values are the mean ± S.E.M. (n = 5). *p < 0.05 in the acrolein-infused SN compared with the control SN by *t*-test. (**E**) A behavioral study of apomorphine-induced rotations in rats subjected to a unilateral infusion of acrolein (n = 6) or vehicle (n = 4) in the SN. Values are the mean ± S.E.M. *p < 0.05 in the contralateral rotations compared with the ipsilateral rotations in rats with acrolein-infused SN by Mann-Whitney Rank Sum Test. n.s.: not significant.

To study the role of acrolein in the pathophysiology of Parkinson’s disease, acrolein (15, 50, 150 nmoles) was locally infused in the SN of chloral hydrate-anesthetized rats. Seven days after the intranigral infusion of acrolein, several neurodegenerative features in the nigrostriatal dopaminergic system of rat brain were demonstrated. First, compared with the dopamine levels in the control striatum, higher doses of acrolein (50 and 150 nmoles) significantly reduced the dopamine content in the striatum ipsilateral to the acrolein-infused SN (Fig. 1A). Furthermore, acrolein dose-dependently decreased tyrosine hydroxylase (TH) and dopamine transporter levels (two biomarkers of dopaminergic neurons) in the infused SN (Fig. 1B,C). At the same time, the immunofluorescent staining study demonstrated acrolein (150 nmoles)-induced reduction in TH-positive neurons in the infused SN (Fig. 1D). Behaviorally, rats subjected to a unilateral infusion of acrolein in SN rotated ipsilaterally to the infused SN when challenged with apomorphine, indicating asymmetric levels of striatal dopamine contents in rat brain (Fig. 1E). These data suggest that intranigral infusion of acrolein induced neurodegeneration of nigrostriatal dopaminergic system of rat brain.

### Acrolein-induced oxidative stress and protein aggregation in nigrostriatal dopaminergic system

**Figure 2:**
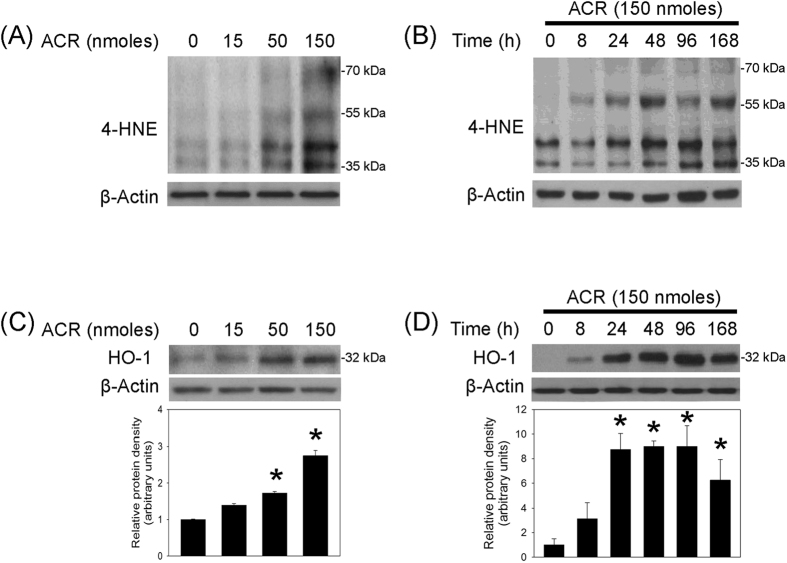
Intranigral infusion of acrolein induced oxidative stress in the nigrostriatal dopaminergic system of rat brain. (**A**) and (**C**): For a dose-dependent effect, ARC (15–150 nmoles) was locally infused in the substantia nigra (SN) and rats were sacrificed 7 days after intranigral infusion of acrolein. (**B**) and (**D**): For a time-dependent effect, acrolein (150 nmoles) was locally infused in the SN and rats were sacrificed at the time indicated. Western blot assay was employed to detect 4-HNE (**A**,**B**) and HO-1 (**C**,**D**) in the SN. Each lane contained 25 μg proteins for all experiments. Graphs show statistical results from relative optical density of bands on the blots estimated by Image J. Values are the mean ± S.E.M. (n = 3/group). *p < 0.05 in the acrolein-infused SN compared with the intact SN by one-way analysis of variance (one-way ANOVA) and followed by the LSD test as a post-hoc analysis.

**Figure 3:**
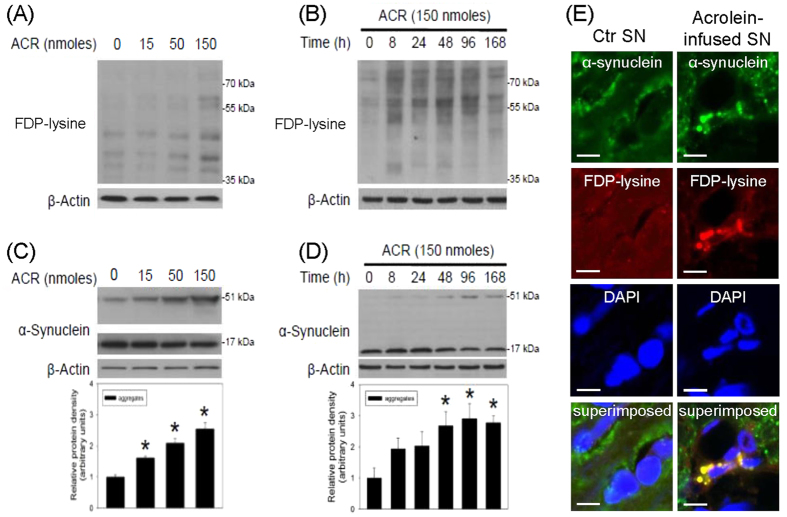
Intranigral infusion of acrolein induced protein conjugation and α-synuclein aggregation in the nigrostriatal dopaminergic system of rat brain. (**A** and **C**): For a dose-dependent effect, ARC (15–150 nmoles) was locally infused in the substantia nigra (SN) and rats were sacrificed 7 days after intranigral infusion of acrolein. (**B** and **D**): For a time-dependent effect, acrolein (150 nmoles) was locally infused in the SN and rats were sacrificed at the time indicated. Western blot assay was employed to detect FDP-lysine (**A**,**B**) and α-synuclein (**C**,**D**) in the SN. Each lane contained 25 μg proteins for all experiments. Graphs show statistical results from relative optical density of bands on the blots estimated by Image J. Values are the mean ± S.E.M. (n = 3/group). *p < 0.05 in the acrolein-infused SN compared with the intact SN by one-way analysis of variance (one-way ANOVA) and followed by the LSD test as a post-hoc analysis. (**E**) Representative confocal microscopic data showed co-localization of FDP-lysine and α-synuclein immunoreactivities in the infused SN of rat brain. Acrolein (150 nmoles) was locally infused in the SN and rats were sacrificed 48 hrs after intranigral infusion of acrolein. Scale bars: 10 μm. Results were repeated in independent experiments.

To investigate the involvement of oxidative stress in acrolein-induced neurotoxicity in the nigrostriatal dopaminergic system, both dose- and time-dependent effects of acrolein on 4-hydroxy-2-noneal (4-HNE, a by-product of lipid peroxidation) and heme oxygenase-1 levels (HO-1, a redox-regulated protein) were measured using Western blot assay. Seven days after the intranigral infusion of acrolein, dose-dependent increases in 4-HNE and HO-1 levels were observed in the acrolein-infused SN (Fig. 2A and C). Furthermore, intranigral infusion of acrolein (150 nmoles) increased 4-HNE and HO-1 levels in a time-dependent manner (Fig. 2B and D). These data indicate that intranigral infusion of acrolein is capable of inducing oxidative stress. Due to the electrophilic property of acrolein, we detected elevations in acrolein-protein adducts by increasing FDP-lysine levels in the acrolein-infused SN (Fig. 3A and B). At the same time, the ability of acrolein to induce α-synuclein aggregation was evaluated by measuring the levels of α-synuclein aggregates. Western blot assay showed that acrolein reduced α-synuclein monomer (17 kDa) and increased α-synuclein trimer levels (51 kDa) in time- and dose-dependent manners (Fig. 3C and D). Furthermore, the immunostaining study demonstrated co-localization of immunoreactivities of acrolein and α-synuclein in the acrolein-infused SN (Fig. 3E). These data indicate that acrolein is capable of inducing protein conjugation and α-synuclein aggregation in the nigrostriatal dopaminergic system of rat brain.

### Acrolein-induced neuroinflammation in nigrostriatal dopaminergic system

**Figure 4:**
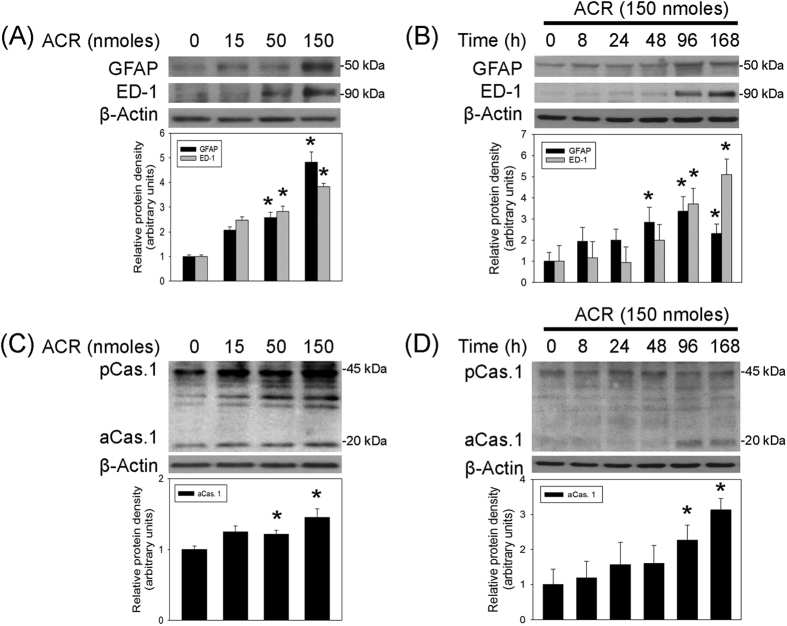
Intranigral infusion of acrolein induced neuroinflammation in the nigrostriatal dopaminergic system of rat brain. (**A** and **C**), For a dose-dependent effect, ARC (15–150 nmoles) was locally infused in the substantia nigra (SN) and rats were sacrificed 7 days after intranigral infusion of acrolein. (**B** and **D**): For a time-dependent effect, acrolein (150 nmoles) was locally infused in the SN and rats were sacrificed at the time indicated. Western blot assay was employed to detect GFAP and ED-1 (**A**,**B**) as well as caspase 1 activation (**C**,**D**) in the SN. Each lane contained 25 μg protein for all experiments. Graphs show statistic results from relative optical density of bands on the blots estimated by Image J. Values are the mean ± S.E.M. (n = 3/group). *p < 0.05 in the acrolein-infused SN compared with the intact SN by one-way analysis of variance (one-way ANOVA) and followed by the LSD test as a post-hoc analysis.

The involvement of neuroinflammation in acrolein-induced neurotoxicity was investigated by measuring GFAP and ED-1, respective biomarkers of activated astrocytes and microglia. Intranigral infusion of acrolein induced time- and dose-dependent increases in GFAP and ED-1 in the acrolein-infused SN (Fig. 4A and B). Furthermore, the effect of acrolein on inflammasome formation (a central role in neuroinflammation) was studied by measuring active caspase 1, an inflammatory caspase of inflammasome. Western blot assay showed that acrolein increased active caspase 1 levels (20 kDa) in the infused SN (Fig. 4C and D). These data suggest that acrolein is capable of inducing neuroinflammation, including glial activation and inflammasome formation.

### Acrolein-induced programmed cell death in nigrostriatal dopaminergic system

**Figure 5:**
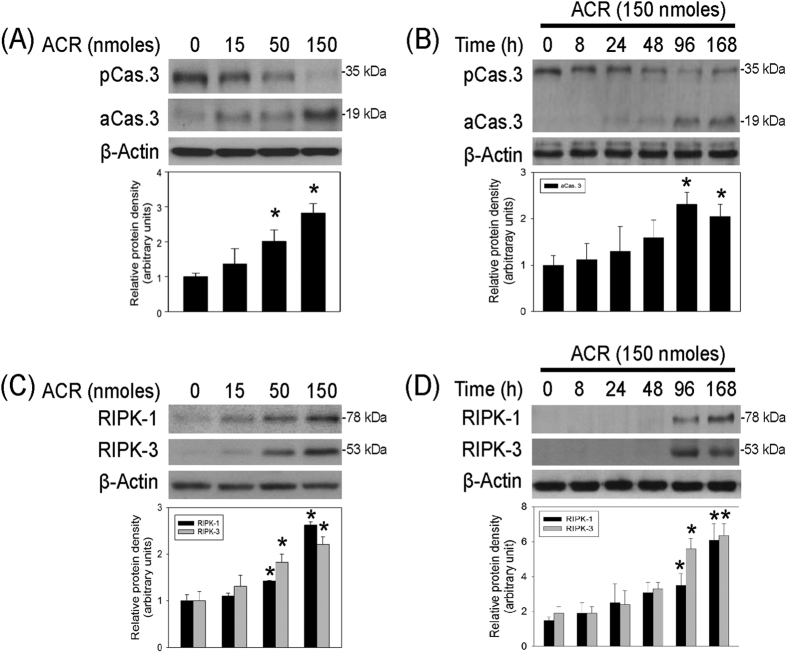
Intranigral infusion of acrolein induced programmed cell death in the nigrostriatal dopaminergic system of rat brain. (**A** and **C**), For a dose-dependent effect, ARC (15–150 nmoles) was locally infused in the substantia nigra (SN) and rats were sacrificed 7 days after intranigral infusion of acrolein. (**B** and **D**): For a time-dependent effect, acrolein (150 nmoles) was locally infused in the SN and rats were sacrificed at the time indicated. Western blot assay was employed to detect caspase 3 activation (**A**,**B**) as well as RIP-1 and RIP-3 (**C**,**D**) in the SN. Each lane contained 25 μg protein for all experiments. Graphs show statistic results from relative optical density of bands on the blots estimated by Image J. Values are the mean ± S.E.M. (n = 5/group for A, n = 3/group for **B**–**D**). *p < 0.05 in the acrolein-infused SN compared with the intact SN by one-way analysis of variance (one-way ANOVA) and followed by the LSD test as a post-hoc analysis.

The neurotoxic mechanisms underlying acrolein-induced neurodegeneration were investigated by measuring the incidences of apoptosis and necroptosis. Seven days after intranigral infusion of acrolein, dose-dependent reduction in procaspase 3 levels (35 kDa) and elevation in activated caspase 3 levels (19 kDa, a hallmark of apoptosis) were observed in acrolein-infused SN (Fig. 5A). Furthermore, acrolein dose-dependently increased receptor interacting protein kinases (RIPK)-1 (78 kDa) and RIPK-3 levels (53 kDa), biomarkers of necroptosis, in the infused SN (Fig. 5B). For a time-dependent effect, acrolein (150 nmoles) significantly increased active caspase 3, RIPK-1 and RIPK3 levels from 96–168 hrs after intranigral infusion (Fig. 5C and D). These data indicate that both apoptosis and necroptosis were involved in the acrolein-induced neurotoxicity in nigrostriatal dopaminergic system of rat brain.

## Discussion

To study the neurodegeneration of nigrostriatal dopaminergic system in Parkinson’s disease, several well-known neurotoxins, including rotenone and 1-methyl-4-phenyl-1,2,3,6-tetrahydropyridine have been used *in vitro* and *in vivo*^27^. In the present study, we are the first to employ intranigral infusion of acrolein which better mimics the situation in PD patients as it accumulates in these patients^8^, while other Parkinsonian neurotoxins, including MPTP, do not. In the past, local infusion of acrolein has been used in a spinal cord injury animal model by infusing 1.6 μl of 1 M acrolein (equivalent to 1.6 μmole)^28^. Here, we infused a smaller volume (0.5 μl) of lower concentrations of acrolein (0.03–0.3 M) for the PD model. The acrolein doses we used were 15–150 nmoles which were only 1–10% of that in the spinal cord injury animals^28^. Using this PD model, our data are the first to show that acrolein is capable of inducing neurodegeneration of nigrostriatal dopaminergic system of rat brain, including acrolein-induced reductions in TH and dopamine transporter levels, decreases in TH-positive neurons in the acrolein-infused SN, depletion in striatal dopamine levels and apomorphine-induced asymmetric rotation. These *in vivo* results demonstrate a PD animal model of acrolein-induced neurodegeneration of nigrostriatal dopaminergic system of rat brain.

Due to its reactive properties, acrolein reportedly plays a pro-oxidative role in the CNS neurodegenerative diseases^25^. In support of this notion, we demonstrated that acrolein increased 4-HNE levels, indicating that acrolein initiated lipid peroxidation^29^ and thus elevated oxidative stress in the nigrostriatal dopaminergic system of rat brain. HO-1 is known to increase under oxidative stress^30^. Our previous studies have shown elevations in HO-1 expression in response to oxidative stress by iron and arsenite^31^,^32^. *In vitro* studies have reported acrolein-increased HO-1 protein expression in the endothelial cells^33^. Consistently, our *in vivo* data showed acrolein-induced elevation in HO-1 levels in the infused SN of rat brain, suggesting that acrolein is pro-oxidative and is capable of inducing oxidative injury.

Because of its electrophilic activity, acrolein reportedly attacks biomolecules, including proteins^4^,^17^. In the present study, we detected significant increases in FDP-lysine adducts i.e., acrolein-lysine modified proteins in the infused SN. Furthermore, we chose α-synuclein as a target protein for acrolein and found co-localization of acrolein and α-synuclein in the infused SN. These data further support the clinical finding of acrolein-α-synuclein conjugates in the SN of PD patients^8^. Moreover, we are the first to show that acrolein is capable of inducing α-synuclein aggregation in the infused SN, suggesting a pathological role of acrolein in forming α-synuclein aggregates detected in the Lewy bodies of PD brain^22^. Accordingly, acrolein may exert its neurotoxic action via conjugating proteins, altering protein conformation, forming misfolding proteins and protein aggregates in the affected brain tissues.

Oxidative stress, α-synuclein aggregation and cell death are suggested previously as a vicious cycle in the pathophysiology of Parkinsonism; neuroinflammation may be the center of this vicious cycle^34^ in acrolein-induced neurotoxicity. Indeed, we found that acrolein is pro-inflammatory by activating astrocytes and microglia. Activated glial cells are important sources of pro-inflammatory cytokines and free radicals^35^,^36^ which reportedly contribute to α-synuclein aggregation^37^. In this PD model, acrolein may exert its neurotoxic action by deregulating inflammasome activity^38^ in the activated microglia^39^. The first line of evidence for acrolein-induced inflammasome formation is caspase1 activation in the acrolein-infused SN. One possible mechanism for acrolein-induced inflammasome formation may be due to acrolein-induced α-synuclein aggregation^40^. Several pathological functions of caspase 1 have been suggested. First, caspase 1 has been shown to induce α-synuclein aggregation^41^ and thus may augment the vicious cycle in the affected nigrostriatal dopaminergic system. In addition, caspase 1 is known to proteolytically cleave pro IL-1β into the biologically active IL-1β, an pro-inflammatory cytokine^42^,^43^. In support of this notion, we detected reduction in proIL-1β and elevation in mature IL-1β levels in the acrolein-infused SN (data not shown).

Acrolein-induced neurotoxicity is reportedly mediated via non-programmed necrosis and apoptosis^16^,^23^,^44^. Consistently, we detected acrolein-induced apoptosis because intranigral infusion of acrolein activated caspase 3 in the infused SN. In addition, our data are the first to suggest two types of programmed necrosis involved in the acrolein-induced neurotoxicity in nigrostriatal dopaminergic system of rat brain. One is necroptosis, a kinase-regulated necrotic process involving RIPK in the necrosome complex^45^. Acrolein-induced elevations in RIPK-1 and RIPK-3 suggest that acrolein is capable of inducing necroptosis which results in rapid plasma membrane permeabilization, release of cell contents and exposure of damage-associated molecular patterns^45^. The other is pyroptosis, an inflammation-related cell death^46^,^47^. During the acrolein-induced inflammasome formation, activated caspase 1 may cleave gasdermin D and then result in pyroptosis, a lytic cell death^46^,^47^.

In conclusion, we establish a PD animal model by intranigral infusion of acrolein and show that acrolein is neurotoxic via first inducing oxidative stress (8–24 h), protein conjugation (8–24 h), and then α-synuclein aggregation (48 h) and neuroinflammation (48 h) in the nigrostriatal dopaminergic system of rat brain. Later on, acrolein induces apoptosis, necroptosis and pyroptosis (96 h) which finally leads to neurodegeneration of nigrostriatal dopaminergic system of rat brain.

## Materials and Methods

Adult, male Sprague-Dawley rats, weighing 250–350 g, were purchased from by the National Laboratory Animal Breeding and Research Center, Taipei, Taiwan, R.O.C. All animals were housed in an air-conditioned room (22 ± 2 °C) on a 12-h light/dark cycle (06:00–18:00 h) and had free access to food and water. The use of animals has been approved by the Institutional Animal Care and Use Committee of Taipei Veterans General Hospital, Taipei, Taiwan, R.O.C. The methods were carried out in accordance with the approved guidelines. All experiments were performed in the accordance with relevant guidelines and regulation. The approval number is IACUC2014–186.

### Intranigral infusion of acrolein

Rats were anesthetized with chloral hydrate (450 mg/kg, i.p., Sigma, St. Louis, MO) and placed in a stereotaxic instrument (David Kopf Instruments, Palo Alto, CA). After skin incision and exposure of the parietal bone, holes were drilled above the cortical surface for intranigral infusion of drugs. Acrolein (Sigma, St. Louis, MO) prepared in filtered normal saline was infused stereotaxically into substantia nigra (SN) according to the following coordinates (Paxinos and Watson, 1986): 3.2 mm anterior, 2.1 mm lateral to the midline 2 mm above the interaural zero, and 3.5 mm below the incisor bar. Acrolein prepared in saline (0.5 μl) was infused at a rate of 0.2 μl/min through a 30 gauge stainless steel needle. The injection needle was held in place for an additional 3 min following drug infusion. After surgery, rats recovered from anesthesia. For the dose-dependent effect of acrolein, 3 groups of rats (3–5 rats/group) were used for intranigral infusion of acrolein (15, 50, 150 nmoles). Rats were sacrificed 7 days after intranigral infusion of acrolein. For the time-dependent effect of acrolein, 5 groups of rats (3 rats/group) were used. Rats were sacrificed 8, 24, 48, 96, 168 h after intranigral infusion of acrolein (150 nmoles). The intact SN in the contralateral hemisphere was used as a control group to the acrolein-infused SN^48^,^49^.

### HPLC-EC analysis of striatal dopamine content

After decapitation, striata were dissected and immediately frozen in liquid nitrogen and stored at −70 °C until analysis. An HPLC coupled with electrochemical detection procedure was used to quantify dopamine content in striatum.

### Immunofluorescence staining

Forty-eight hours after intranigral infusion of acrolein, rats were deeply anesthetized and then perfused transcardially using 4% paraformaldehyde in 0.1 M PBS. Brains were removed and placed in 30% sucrose-PBS overnight and frozen-sectioned coronally at 30 μm thickness. Sections were washed with 0.1 M PBS, incubated with 0.3% Triton X-100 and 1% goat serum (GS; Sigma, St. Louis, MO), and blocked with 3% GS for 60 min. Sections were then incubated overnight at 4 °C with primary antibodies specific for TH (Cell Signaling Tech., Danvers, MA), α-synuclein (Abcam, Cambridge, UK) and FDP-lysine (Abcam, Cambridge, UK). Afterwards, sections were incubated for 1 h at room temperature with secondary antibodies conjugated with rhodamine and fluorescein isothiocyanate (Millipore Corporation, Billerica, MA,). Nuclei were labeled with 4′, 6-diamidino-2-phenylindole (1 mg/ml) for 10 min at room temperature. Sections were mounted in glycerol and visualized by a fluorescence confocal microscope (FluoView, Olympus, Tokyo, Japan). TH-positive cells in acrolein-infused SN were counted manually and expressed as % of that in the contralateral intact SN.

### Western blot analysis of relevant proteins

Frozen SN was homogenized with a sonicator in 40 μl ice-cold protease inhibitor cocktail (Calbiochem, San Diego, CA). After homogenization, the lysates were centrifuged at 12,000 × g for 30 min at 4 °C, the supernatant was frozen in liquid nitrogen and stored at −80 °C. Protein samples (3–25 μg) were run on 8–15% sodium dodecyl sulfate (SDS)-polyacrylamide gel electrophoresis and then transferred onto a nitrocellulose membrane (Bio-Rad, Hercules, CA) at 80 V for 120 minutes. Blots were probed with a monoclonal antibody against tyrosine hydroxylase (TH) (1:3000, Chemicon, Temecula, CA), dopamine transporter (1:1000, Millipore, Bedford, MA), 4-hydroxy-2-noneal (4-HNE)(1:1000, Abcam, Cambridge, UK), heme-oxygenase-1 (HO-1) (1:2000, StressGen, Victoria, CA), N-(3-formyl-3,4-dehydropiperidino)lysine (FDP-lysine, an epitope for antibody against acrolein) (1:500, Abcam, Cambridge, UK), α-synuclein (1:1000, BD Transduction Lab., Lexington, KY), glial fibrillary acidic protein (GFAP) (1:3000, Millipore, Bedford, MA), ED-1 (1:1000, AbDSerotec, Raleigh, NC), caspase-1 (1:1000, Santa Cruz Biotech, Santa Cruz, CA), caspase-3 (1:1000, Cell Signaling Tech., Beverly, MA), RIPK-1 and RIPK-3 (1:1000, Cell Signaling Tech., Danvers, MA) at room temperature for 2 h. After primary antibody incubation, the membrane was washed and incubated with horseradish peroxidase-conjugated secondary IgG (1:3000; Chemicon, Temecula, CA) for 1 h at room temperature. The immunoreaction was visualized by Amersham enhanced chemiluminescence (Amersham Pharmacia Biotech, UK). After this detection, the bound primary and secondary antibodies were stripped by incubating the membrane in stripping buffer (100 mM 2-mercaptoethanol, 2% SDS) at 50 °C for 45 min. The membrane was reprobed with β-actin antibody (1:5000, Chemicon, Temecula, CA).

### Behavioral study: apomorphine-induced rotation

Two groups of rats were used, one received intranigral infusion of acrolein (150 nmoles), the other received vehicle (saline). Seven days after intranigral infusion, rats were treated with apomorphine (1 mg/kg, s.c.)^50^,^51^ Apomorphine-induced rotations were monitored and recorded by a video camera. The extent of acrolein-induced neurotoxicity is presented as the number of contralateral turns for 60 mins after apomorphine injection.

### Statistics

All data are expressed as the mean ± S.E.M. Statistical comparisons were made using *t*-test for dopamine transporter and Mann-Whitney rank sum test for apomorphine-induced rotation. Statistical comparisons of the rest data were made using one-way analysis of variance (one-way ANOVA) and followed by the LSD test for the rest results as a post-hoc analysis. A value of p < 0.05 was considered significant.

## Additional Information

**How to cite this article**: Wang, Y.-T. *et al*. Acrolein acts as a neurotoxin in the nigrostriatal dopaminergic system of rat: involvement of α-synuclein aggregation and programmed cell death. *Sci. Rep.* **7**, 45741; doi: 10.1038/srep45741 (2017).

**Publisher's note:** Springer Nature remains neutral with regard to jurisdictional claims in published maps and institutional affiliations.
